# Retroperitoneal Ancient Neurilemmoma: A Nervous Rarity

**DOI:** 10.7759/cureus.28940

**Published:** 2022-09-08

**Authors:** Vaidehi Mendpara, Sweta Sahu, Devarsh N Shah, Subhangi Parmar, Tanveer Ahamad Shaik, Swastika Sedhai, Mukesh Pancholi

**Affiliations:** 1 Surgery, Government Medical College, Surat, Surat, IND; 2 Surgery, Jagadguru Jayadeva Murugarajendra (JJM) Medical College, Davanagere, IND; 3 Medicine and Surgery, Medical College, Baroda, Vadodara, IND; 4 Surgery, Gujarat Cancer Society (GCS) Medical College, Ahmedabad, IND; 5 Cardiovascular Medicine, University of Louisville School of Medicine, Louisville, USA; 6 Medicine, Kathmandu University, Kathmandu, NPL

**Keywords:** rare, general surgery, retroperitoneal tumor, benign tumour, neurilemmomas

## Abstract

Neurilemmomas are tumors of neural origin that comprise Schwann cell proliferation in a characteristic pattern. They are benign in nature. Ancient neurilemmomas are usually longstanding growths that exhibit degenerative features that could be mistaken for malignancy. We report a case of ancient neurilemmoma in a 70-year-old male patient in the retroperitoneal area. Retroperitoneal schwannomas are extremely uncommon along with ancient neurilemmoma features making it worth reporting.

## Introduction

Ancient neurilemmomas are tumors of a rare category. These originate from peri-neural Schwann cells. The term “ancient” denotes degenerative features of hemorrhage, cyst formation, hyalinization, and calcification acquired due to advancing age in these tumors [1]. These are incidentally detected or have local symptoms due to their benign and slow-growing nature. The majority of schwannomas tend to be benign, but around 5-18% of cases are malignant and are associated with von Recklinghausen disease. The retroperitoneal location of schwannomas makeup 0.5-3% of all cases and hence is a rarity [2]. Percutaneous needle biopsy or core biopsy aids in the diagnosis of these patients as the tumors are encapsulated and in the retroperitoneum. Complete resection of the tumor is curative in most patients, and the reappearance of the tumor after complete surgical resection is rare [1].

## Case presentation

Clinical findings

A man aged seventy years presented with chief complaints of fever and headache for a few days. He also had a complaint of lower back and bilateral limb pain for four months. The routine physical examination was normal, and the abdominal examination showed no positive findings. The blood examination reports revealed that the patient was found to be positive for malaria (falciparum) and dengue, which explained the constitutional symptoms. However, chronic bilateral lower limb and lower back pain prompted the further radiological investigation. The patient was started on antimalarials and treatment for dengue fever along with conservative management of pain and radiologic investigation during the first admission showed mild disc bulge along with an incidental mass in the left retroperitoneal region. The patient was kept under observation for the incidental radiologic findings till the management of the diagnosed infection. The patient was discharged after treatment for malaria and dengue and came for a follow-up visit after one month with persistent abdominal pain radiating to the back that was not relieved by pain medication prescribed during the first admission. Hence, radiologic investigation during this visit showed a growing mass, and the patient was admitted for evaluation and surgical removal.

Radiologic findings

**Figure 1:**
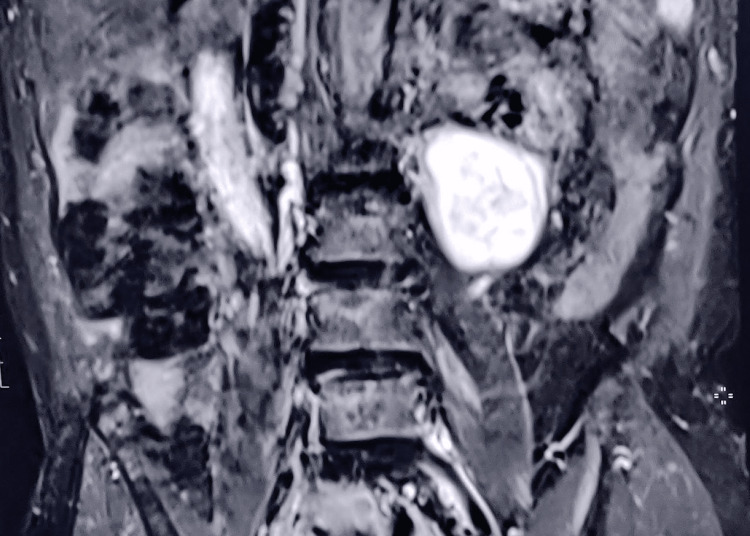
Magnetic resonance imaging of the lumbar coronal section showing a mass lesion in the left paravertebral region.

Ultrasonography (USG) of the abdomen was ordered during the first admission to rule out hepatosplenomegaly but showed no positive findings. However, USG findings rather showed an approximately 5.5 × 4.3 cm sized heterogeneous echotexture lesion, predominantly hypoechoic with a few anechoic areas in it with minimal internal vascularity just medial to the lower pole of the left kidney in the retroperitoneal region. An MRI scan was also ordered during the first admission showing marginal peridiscal osteophytes at the lumbar vertebra with subtle Modic type-2 regenerator changes at adjacent cortical plates and diffuse disc bulge with left paracentral disc extrusion at the L5-S1 level, causing compression over the left transverse nerve root along with mild diffuse disc bulge at L2-L3 level without significant neural compression. The scans also showed a well-defined incidental heterogeneous lesion measuring 4.8 × 4.8 cm seen adjacent to the left anterior surface of the left psoas muscle in the left retroperitoneal space. During the follow-up visit after one month for the incidental radiologic findings, evaluation on USG showed an increase in the size of the retroperitoneal lesion to approximately 6.4 × 4.3 cm having mixed echogenic lesion with a few anechoic cystic areas, in its suggestive of necrosis with minimal internal vascularity and foci of calcification noted in the para-aortic region on the left side adjacent to the lower pole of the left kidney (Figure 1).

Biopsy findings 

**Figure 2:**
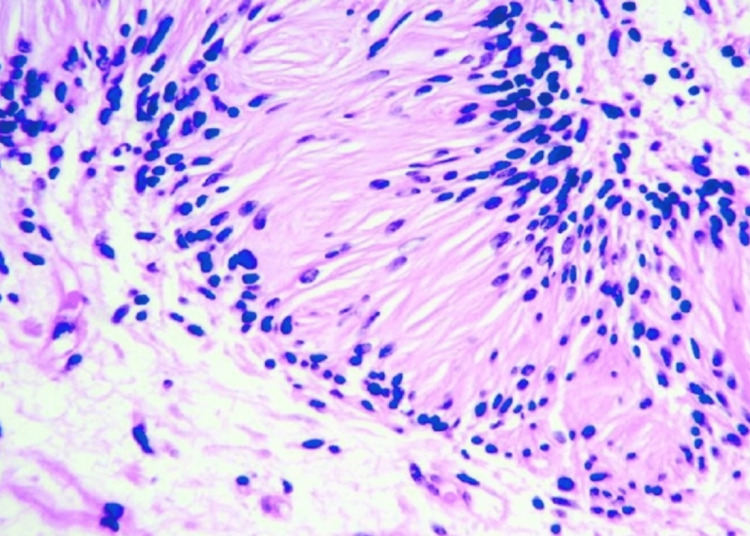
Microscopic view of neurilemmoma showing verocay bodies.

Ultrasonography (USG) guided percutaneous tru-cut needle biopsy of the mass was taken during the second admission after one month of the initial incidental finding to further assess the pathology and rule out malignancy. The report suggested predominant fibro-collagenous tissue with a few benign spindle cells along with focal areas of chronic inflammatory infiltrate consisting of lymphocytes likely to be a schwannoma (Figure 2).

Management

**Figure 3:**
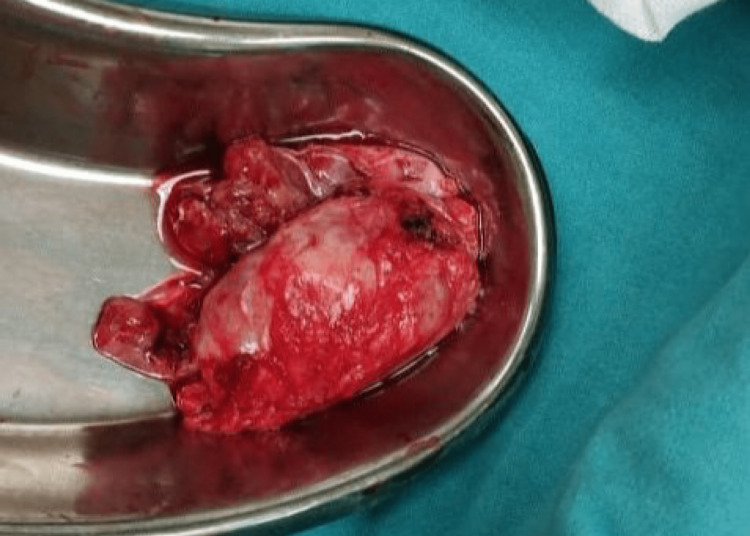
Excised specimen of retroperitoneal mass.

**Figure 4:**
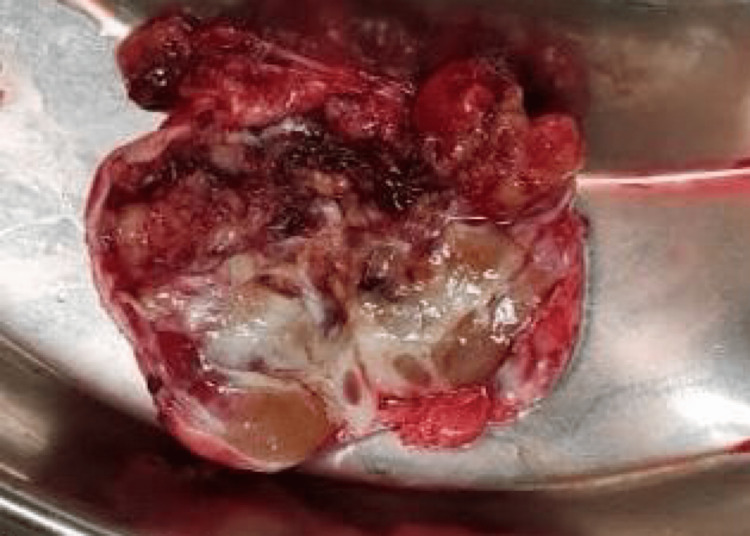
Cut surface of the specimen is showing large cystic regions and areas of hemorrhage and calcification.

Exploratory laparotomy done during the second admission after four days of positive biopsy findings showed a mass medial to the left kidney adjacent to the L2-L3 vertebrae, which was excised en bloc with no signs of invasion in the surrounding tissues. The excised mass was sent for histopathologic examination, whose results confirmed the biopsy findings (Figure 3). Gross histologic and microscopic features were highly suggestive of atypical neurilemmoma (ancient neurilemmoma). The postoperative period was uneventful and the patient was discharged five days after the surgery with advice for weekly follow-up. The patient had significantly reduced pain during his visit for suture removal after two weeks of discharge. USG was done during that visit as well as at the six-month follow-up, which did not show any positive findings suggestive of recurrence. The cut surface of the specimen showed large cystic regions and areas of hemorrhage and calcification (Figure 4).

## Discussion

Retroperitoneal schwannomas are such rare tumors that they make up only about 1-5% of all masses in the retroperitoneal region. The tumor localization in the retroperitoneal region accounts for 0.75-2.6% of all schwannomas, while the majority are localized in the extremities, head and neck. These are usually solid, firm, and solitary, with a well-circumscribed smooth surface. They are large and encapsulated tumors originating from the paravertebral region. Macroscopically, schwannomas tend to undergo spontaneous degeneration with hemorrhage more than those in the head and neck and extremities. Prevalence is higher in the older age group, with patients usually not being diagnosed before the age of 40-60, also the male-female ratio of these tumors is almost similar, which is roughly 2:3 in proportion [3,4]. Ackerman and Taylor originally described ancient schwannomas in 1951, which account for 0.8% of soft tissue tumors. Ancient schwannomas have distinct degenerative features, including cystic necrosis, xanthomatous changes, fibrosis, calcification, stromal edema, perivascular hyalinization, and degenerative nuclei with hyperchromasia, lobulation, and pleomorphism. As the tumor grows with time, there are areas of tumor degeneration due to vascular insufficiency. Regardless of these degenerative changes, a similarity exists between ancient schwannomas and their traditional counterparts, which are benign and slow-growing tumors with rare malignant transformation [1]. Typically, the symptoms are vague and include dull back, flank, and abdominal pain. Urinary symptoms like hematuria increased urination frequency, and colicky pain may also be present in the mass next to the kidneys [5].

The tumor is generally encapsulated and, on microscopy, we mainly find two main components: a cellular region arranged in order (Antoni A area) and a loosely arranged myxoid component (Antoni B area). The cellular regions are sclerosed or fibrotic, and over time, hematomas and cysts develop due to degenerative changes. Nuclear palisades are absent, which are normally present in classic schwannoma. Common findings like nuclear atypia and hyperchromasia were present in this case. Malignancy can be ruled out in the absence of mitoses and the presence of a preserved cohesive clustering of spindle-shaped cells. The neural origin of these tumors results in diffuse positivity on immunostaining with S-100 [3]. One of the hypotheses describes the degenerative characteristics of ancient schwannoma due to the frequently encountered vessels within the Antoni B cells, which are hyalinized leading to ischemic changes and hemorrhage ultimately allowing cyst formation [6]. Pathological, histological, and immunohistochemical findings aid in definitive diagnosis. 

Radiological imaging is important as it helps in guiding treatment plans and also provides information related to tumor size, location, and invasiveness to other structures. Spinal cord tumors or tumors surrounding the sciatic or femoral plexus can cause compression of nerves and give clinical findings similar to herniation of the vertebral disc as seen in this patient. Hence, the radiologic investigation becomes of utmost importance as such tumors are likely to be misdiagnosed as discal herniation, which could delay appropriate management [7]. Ultrasonography can be a very helpful and economical tool for detecting these tumors primarily along with further evaluation via CT scans and MRI. CT scans can be used to identify cystic regions with well-defined low or mixed attenuation and central necrosis. Retroperitoneal variants of schwannomas present more commonly with cystic changes than other retroperitoneal tumors [1]. MRI with gadolinium enhancement is patronized over CT in showcasing cystic tumor degeneration, showing margins and points of neuronal origin. However, radiological findings cannot differentiate a benign mass from a malignant sequel unless the findings show tumor invasion or metastasis [8]. 

Neurofibroma, liposarcoma, pheochromocytoma, paraganglioma, malignant fibrous histiocytoma, hematoma, and lymphangioma are among the difficult differential diagnoses for retroperitoneal schwannomas [4]. There is a prominent likelihood of local recurrence and malignant changes even though it was diagnosed as benign. As a result, total surgical excision is the most effective treatment for retroperitoneal schwannoma since it is difficult to rule out malignancy through preoperative and perioperative excision, necessitating the removal of the entire tumor. A magnified view in laparoscopy makes the resection of tumors easier and more complete compared to open surgery [9]. Controversy persists over surrounding soft tissue margins reporting negative after tumor removal, especially when surrounding viscera or tissue needs to be immolated. Many investigators advise complete excision, if necessary, including the immolation of surrounding tissues, while others suggest that basic enucleation or partial excision is enough.

## Conclusions

Ancient neurilemmoma in the retroperitoneal region is an uncommon benign variant of schwannoma with rare malignant development. In our situation, it was diagnosed incidentally on the radiologic investigation done to evaluate symptoms suggestive of lumbar radiculopathy. Usually, schwannomas are asymptomatic slow-growing tumors but considering the location adjacent to the vertebral column in the retroperitoneum, even a slight increase in size, as was in this case, can cause compression symptoms mimicking radiculopathy. However, since typical symptoms of radiculopathy were not characteristically present and the incidental mass in that very region was increasing in size, it was speculated that the growing tumor was contributing to the patient's chronic presenting symptoms and hence its removal became imperative. The tumor was effectively removed by exploratory laparotomy with no complications and a positive outcome with a significant reduction of pain was anticipated.
